# Expression of Galectin-3 As A Testis Inflammatory Marker in Vasectomised Mice

**Published:** 2013-05-05

**Authors:** Hamed Haddad Kashani, Ghazale Moshkdanian, Mohammad Ali Atlasi, Ali Akbar Taherian, Homayoun Naderian, Hossein Nikzad

**Affiliations:** Anatomical Sciences Research Center, Kashan University of Medical Sciences, Kashan, Iran

**Keywords:** Vasectomy, Galectin-3, Inflammation, Testis, Immunohistochemistry

## Abstract

**Objective:**

Vasectomy, though in some cases are being confronted with irreversibility, has been accepted as an effective contraceptive method. It is estimated that near 2-6% of vasectomised men ultimately show a tendency to restore their fertility. In some cases, vasectomy has been considered as an irreversible procedure due to many post-vasectomy complications causing this debate. The aim of present study was to investigate the pattern of expression of galectin-3, an inflammatory factor secreted by macrophages and immune cells, following the vasectomy in mice testis tissue.

**Materials and Methods:**

In this experimental study, twenty mature male Balb/c mice, aged two months, were divided into two equal groups: sham and vasectomised groups (n=10). They were sacrificed four months after vasectomy, while the pattern of galectin-3 expression was investigated using a standard immunohistochemistry technique on testicular tissues. Stereological analyses of testes parameters in vasectomised and shamoperated groups were compared by mixed model analysis.

**Results:**

Based on observations, although galectin-3 was not expressed in sham-operated group, it was expressed in 40% of testicular tissues of vasectomised mice, like: seminiferous tubules, interstitial tissues and tunica albugina. Also, our result showed a significant alteration in number of germ and sertoli cells of testicular tissue in vasectomised group in comparison to sham-operated group. In addition, the result of mixed model method confirmed a significant reduction in germ and sertoli cells of vasectomised group.

**Conclusion:**

The expression of galectin-3 at different parts of testicular tissue in vasectomised group is higher than sham group. This express illustrates the increase of degenerative changes and inflammation reactions in testicular tissue, leading to chronic complications and infertility, after the vasovasostomy

## Introduction

Vasectomy is a minor surgical procedure, much less complicated than the tubal ligation in women. Among some documents showing the advantages and disadvantages of this procedure, the most prominent advantage is its reversibility, while near 10% of the vasectomised men usually suffer from bleeding, feel pain and inflammation. Inflammation and fibrosis are the most important indicators of inflammatory response, which show some alteration occurred at the suspected testis.

T cell lymphocytes and monocyts are responsible for cell mediated immunity and phagocytosis, respectively. After identifying the antigen, T cell lymphocytes induces the B cell lymphocytes to make antisperm antibodies (2).

Monocyte-monocyte interactions leads to macrophage development (3), which it goes through the phagocytes pathway, depletion of germ cells, inflammation and fibrosis of testis. Some studies have revealed expression of galectin-3 in damaged tissues (4, 5).

Galectin-3 is a β-galactoside-binding lectin of 30 KDa that has been implicated in inflammation and fibrosis (6, 7). Macrophages produce Galectin-3 in large numbers (8), which it causes monocytemonocyte interactions, subsequently leading to multinucleated giant cell development. This phenotype- associated phenomenon is recognized by activation of macrophage (3), chronic inflammatory and fibrotic diseases (9). The condition of upregulation during galectin-3 expression is a feature of the alternative macrophage phenotype in which release of galectin-3 is activated by alternatively activated macrophages. In acute inflammatory injury, macrophage reduction results in generation of fibrosis (10, 11).

The aim of present study was to determine expression of galectin-3 as a marker of inflammation and fibrosis as well as to verify histological changes of testis following the vasectomy in adult mice.

## Materials and Methods

### Animals

In this experimental study, mature Balb/c mice from an inbred colony obtained from the Department of Anatomy located at the Kashan University of Medical Science, were used. Twenty male mice, two months old, were divided into two equal groups comprising 10 animals. They were assigned as sham-operated and vasectomised groups. Both groups were sacrificed four months after vasectomy. Animal experimentation was approved by the Animal Research Committee of Kashan University of Medical Science.

### Surgical procedure

Bilateral vasectomy was performed with sterile precautions. Anesthesia was induced by intraperitoneal injection of 30 mg/kg of pentobarbitone sodium (Somnitol; MTC Pharmaceuticals, Hamilton, Canada), supplemented by ( suggestion: along with) inhalation of ether (12). The ductus deferens was exposed and doubly ligated with 3-0 silk suture, whereas its blood vessels were included in the ligatures. A part of ducts in length of four mm was then excised between the ligatures. Finally, the cremaster muscle and skin were closed with 3-0 chromic catgut and 3-0 silk sutures, respectively, and the wound was protected by plastic dressings. The procedure was duplicated for the sham animals. Briefly, the incision was made, vasa deferentia were exposed, 3.0 silk was passed, but the vasa deferentia were not ligated or transected. The mice were killed by an overdose of ether four months after operation.

### Histology

#### Tissue samples

Testes were removed from vasectomised and sham-operated animals after operation; they were then cleaned, weighted carefully by a Sartorius weighing machine, and compromised with each other. Finally, testes of both groups were fixed in Bouin fixative and embedded in paraffin using standard techniques in order to analyze by a light microscopy. For histological evaluation, 5-µmthick sections were stained with Hematoxylin- Eosin method. All of chemical materials were purchased from Merck (Germany).

### Morphometric procedure

#### Cell counting

Round cross-section seminiferous tubules at VIIVIII cellular cycle stages were selected, randomly. Spermatogonia, spermatocyte I, spermatid, mature sperm and sertoli cells were enumerated using an optical microscope (Zeiss Company, Germany) with a 400x magnification, also the stereological techniques as described by others were applied (13, 14). For each mouse, 10 sections of seminiferous tubules were used, randomly.

#### Seminiferous tubule diameter

We measured diameter of seminiferous tubules and their lumens as well as epithelium thickness of vasectomised and sham-operated groups. The diameters of 10 randomly selected round seminiferous tubules per mouse were measured across the axes of their profiles with an ocular micrometer with 10x magnification, calibrated by means of a stage micrometer (Zeiss Company, Germany).

#### Volumetric composition of testis

The volume densities of seminiferous tubules, tubular lumen and interstitium were determined by point-counting method (14). Briefly, five randomly selected sections per animal in each group were examined using a Zeiss Optical Microscope with 400x magnification, fined with a square lattice containing 121 intersections.

#### VImmunostaining for light microscopy

To study the expression pattern of galectin-3 in testis after vasectomy, immunohistochemistry was performed. For standard immunohistochemical technique, paraffin sections (four µm) were dewaxed in xylen, then were rehydrated in decreasing concentrations of ethanol and distilled water, respectively. Endogenous peroxidase was blocked by 0.3% hydrogen peroxide in methanol for 10 minutes, and in subsequent step, nonspecific antibody binding was blocked by incubation in 5% bovine serum albumin (BSA) in phosphate buffered saline (PBS) for 30 minutes. After this treatment, the sections were washed three times (five minutes each) with PBS and incubated with rabbit polyclonal galectin-3 antibody (Santa Cruz Biotechnology, Inc, Santa Cruz, USA). The antibody was applied at a dilution of 1:100 over night at 4˚C. After washing three times (five minutes each) with PBS, the sections were incubated with horseradish peroxidase (HRP)-donkey anti rabbit antibody (1: 250 diluted) for one hour at room temperature and washed again three times with PBS. Peroxidase activity was revealed by 3, 3’-Diaminobenzidine tetrahydrochloride (DAB)-H_2_O_2_ reaction. The slides were incubated with DAB (0.5 mg/ ml PBS) containing 0.005% H_2_O_2_ for 10 minutes at room temperature, and then washed with distilled water. Finally, slides were counterstained with hematoxylin and mounted. For negative controls, normal rabbit serum IgG was applied as substitute for the primary antibody in the staining protocol. Following preparation, the slides were analyzed and different images were captured.

#### Statistical methods

The obtained data from both groups, expressed as Mean ± SD, were analyzed using the sigma statistical program (version 8.3) and were compared by mixed model analysis. The value of p<0.05 were considered statistically significant.

## Results

**Fig 1:**
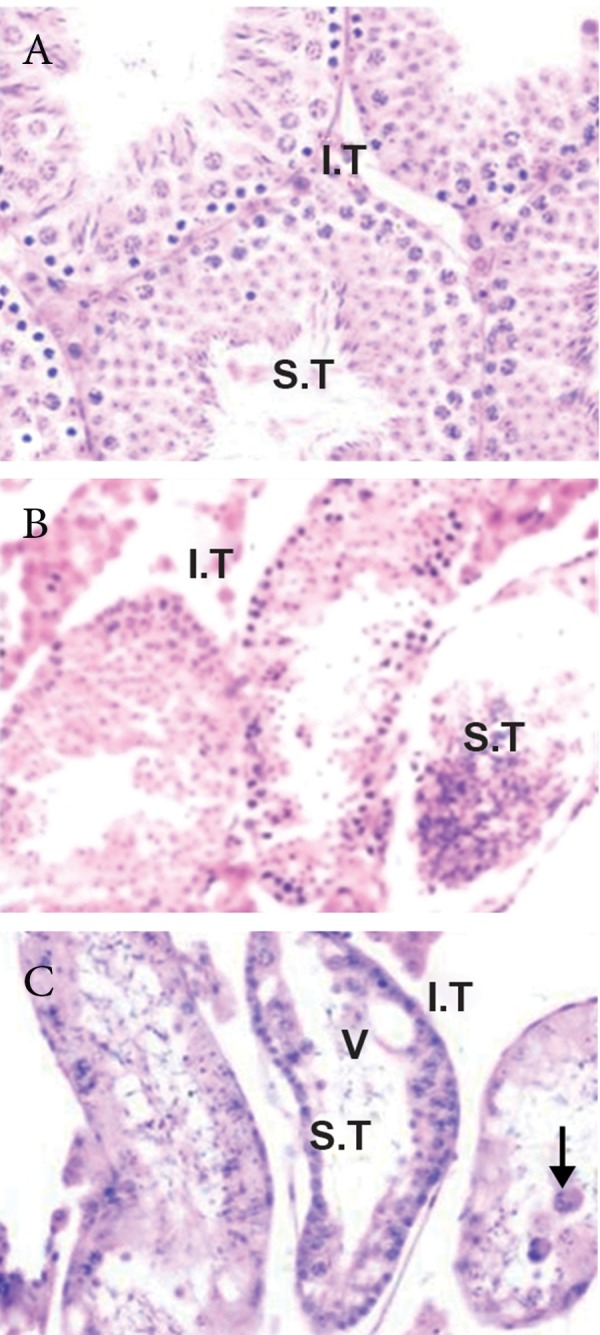
Normal histological features of seminiferous tubules (S.T) and interstitial tissue (I.T) in Sham-operated group (A). Remarkable regressive changes in the seminiferous tubules of the vasectomised group (B, C). These changes included depletion of germ cells, formation of giant cells (arrow), and presence of vacuoles (V) in the epithelium (H&E, × 400).

In histological result revealed a normal histological feature in the testes of sham-operated group. By contrast, remarkable histological changes were observed in the seminiferous tubules in testes of vasectomized group (Fig 1). Four months after vasectomy, some of the seminiferous tubules showed remarkable regressive changes. The changes in the tubules included depletion of germ cells, formation of giant cells, and presence of vacuoles in the epithelium. Sometimes, macrophages ingesting spermatozoa were noticed in the lumen of the seminiferous tubules in testes of vasectomized group.

**Fig 2:**
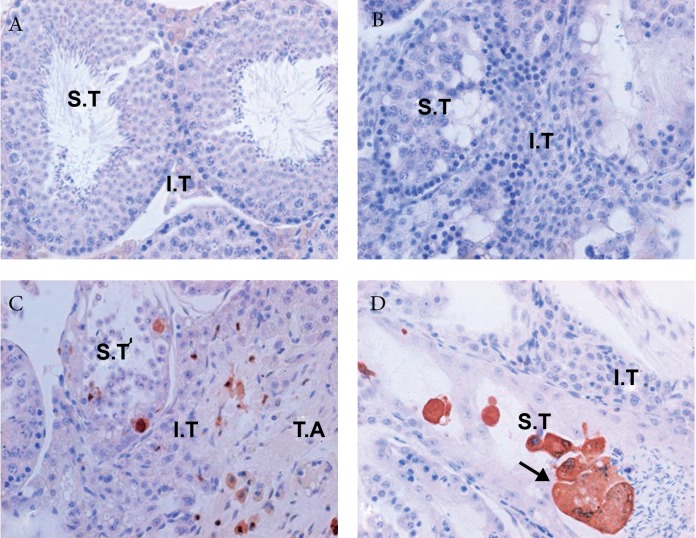
No expression of galectin-3 in testis tissue of sham-operated group (A). No reactivity in the testis tissue of vasectomised group either using the negative control antibody, or in specimens incubated without primary antibody (B). Immunostaining with galectin-3 antibody was showed a high expression of galectin-3 in activated macrophage and immune cells located at degenerated seminiferous tubules (S.T), interstitial tissue (I.T) and tunica albuginate (T.A) of vasectomised group (C). High expression of galectin-3 (arrow) at the site of digestion in sperm cells and giant cells (D) (× 400).

Galectin-3 was not expressed in testis tissue of sham-operated group (Fig 2A), also no reactivity was observed in the testis tissue of vasectomised group either using the negative control antibody, or in specimens incubated without primary antibody (Fig 2B).

Immunostaining with galectin-3 antibody was showed a high expression of galectin-3 in activated macrophages and immune cells located at degenerated seminiferous tubules, interstitial tissue and tunica albuginate of vasectomised group (Fig 2C, D).

Quantitative analysis exhibited a significant reduction in the weight of the testis and thichness of both seminiferous tubules and epithelium in vasectomised group as compared with sham-operated group. But, diameter of lumen was significantly increased (Table 1).

Number of sertoli, spermatocyte I, round spermatid and elongated spermatid cells were significantly decreased in vasectomized group as compared with sham-operated group (Table 2). Number of spermatogonia cells between two groups was not significant.

**Table 1:** Parameters of sham-operated and vasectomised mice


Parameters	Sham-operated(n=10)	Vasectomised(n=10)	P value

**Body weight (g)**	46.02 ± 5.45	45.85 ± 6.08	0.91
**R. Testis wt (g)**	0.14 ± 0.01	0.12 ± 0.01	0.01*
**L. Testis wt (g)**	0.13 ± 0.01	0.11 ± 0.01	0.01*
**Interstitial tissue (Vv%)**	38.08 ± 7.41	42.00 ± 10.30	0.22
**Epithelium (Vv%)**	42.78 ± 4.65	37.14 ± 6.37	0.06
**Lumen (Vv%)**	18.94 ± 3.83	21.22 ± 6.70	0.2
**Lumen diameter (µm)**	78 ± 25	82 ± 20	0.007*
**Epithelium thickness (µm)**	108 ± 20	104 ± 4	0.02*
**Seminiferous diameter (µm)**	188 ± 21	190 ± 21	0.3


* Significant. Data were showed as a mean ± SD.

**Table 2:** Germ cells and sertoli cells were compared between sham-operated and vasectomised groups


Parameters	Sham-operated(n=10)	Vasectomised(n=10)	P value

**Sertoli (No./tubule)**	6.48 ± 2.16	5.09 ± .85	0.019*
**Spermatogonia (No./tubule)**	52.73 ± 16.62	44.77 ± 7.48	0.13
**Spermatocyte I (No./tubule)**	44.61 ± 14.71	34.09 ± 14.71	0.029*
**Round Spermatid (No./tubule)**	317.85 ± 97.81	232.85 ± 68.74	0.012*
**Elongated Spermatid (No./tubule)**	112.60 ± 36.36	65.74 ± 36.36	0.00*


* Significant. Data were showed as a mean ± SD.

## Discussion

This is the first study reporting expression of galectin-3 as a testis inflammatory marker in vasectomised mice. We recognized expression of galectin-3 in different parts of testis tissue, which it indicated inflammatory reactions following vasectomy. Inflammation is a consequence of immunity response occurred at the site of lesion. In our study, galectin-3 was detected mostly at degenerated and depleted seminiferous tubules. Macrophages produce Galectin-3 in large numbers (8), which it causes monocyte–monocyte interactions, subsequently leading to multinucleated giant cell development. This phenotype-associated phenomenon is recognized by activation of macrophage (3), chronic inflammatory and fibrotic diseases (9). Some researchers have shown that alternative activation of macrophages with IL-4 and IL-13 motivates expression of galectin-3 (15). IL-4/IL 13-activated macrophages lead to up regulation of some genes engaged in the mechanisms of fibrosis (16-18) and they produce fibronectin and other matrix proteins(19, 20).

Macrophages are involved in all stages of inflammatory process including fibrosis, tissue repair, and healing (21, 22). On the contrary, diminution during revitalization results in a collapse of resolution due to presence of fibrotic response. Thus, macrophages play distinct roles in injury and repairing. While sperms are destroyed by macrophages and lymphocytes, sperm constituents induce activity of antigen presenting cells, which subsequently start the release of cytokines, leading to a chronic inflammatory pathway. Histiocytes are considered to be as the primary cell in the lesions in the response to the flow of sperm in order to support large number of phagocytic cells in process of sperm absorption. Several studies show the association between alternative macrophage activation and enhanced fibrosis (11, 23). In addition, other researches have revealed that mice with galectin-3-deficiency face a reduced fibrotic phenotype (19), while other studies have illustrated the relation of galectin-3 expression with worse result within myocardial fibrosis (7). Although, some studies have showed that Galectin-3 expression is decreased in tissue fibrosis in diabetic nephropathy (24) and in asthma (25). Following these results, we are able to introduce galectin-3 expression as a chronic inflammatory indication, occurring following of vasectomy; in addition, it causes low chance of vasovasostomy. Additional multicentre studies are required to evaluate the appropriateness and effectiveness of this experiment.

Our results also showed testicular degeneration, germ cells depletion, atrophy of seminiferous tubules, as well as depletion and denudation of tubular epithelium. some articles explain that vasectomy has no deleterious effect on the testis, and the testicular alterations observed after vasectomy are caused by procedural artifacts, such as infection and circulatory disturbances (26). On the other hand, it has been reported that severe tubular atrophy happened during closed technique of vasectomy can destroy the construction of the testis (I made this changes in this sentence to show how to avoid plagiarism) (27). Also, a bilateral deficiency in secretory function of sertoli cell in unilaterally vasectomised dogs, resulting in impaired bilateral spermatogenesis and sperm maturation (28).

Therefore, it is possible to postulate that, following vasectomy, mechanical factors may be the primary cause of atrophy (27, 29), and a lymphocytic response supervenes in an already damaged testis (30). Also, obstruction of the vas deferens, occurring in vasectomy, promotes hydrostatic pressure in the testis and epididymis, directed to testicular alterations (27, 31). Lymphocytic infiltration of the seminiferous tubules and of the interstitium has been mentioned as a specific alteration following vasectomy in guinea pig (30). Testicular changes, detected subsequent to vasectomy, have also been explained as a result of immunological response (32).

Different studies conducted by scientists have revealed the reduction of sertoli cells in postvasectomised men is associated with sertoli cell dysfunction (31-34). Interstitial fibrosis can also influence the paracrine functions of the seminiferous tubules after vasectomy (34), causing to reduce spermatogenesis. Fibrosis could also be the outcome of inflammation, and inflammatory reactions have been detected following vasectomy (35). In our studies, there was significant reduction in the sertoli cell number; furthermore, we noticed a marked reduction in the number of round mature sperms, spermatids and spermatocyte I.

Although most other studies reported no significant alteration in testicular weight following vasectomy (1, 36), our data showed significant reduction in testicular weight following vasectomy. Sackler et al. have also found a significant decrease in the testis weight of vasectomised rats (29).

## Conclusion

The expression of galectin-3 at different parts of testicular tissue in vasectomised mice is significantly higher than sham group. This express increases degenerative changes and inflammation reactions in testicular tissue after vasectomy, leading to chronic complications and infertility, even after the vasovasostomy.
